# *Tetrix*: A novel Tetris-based paradigm for neuroimaging research and clinical applications

**DOI:** 10.3758/s13428-026-03150-6

**Published:** 2026-08-24

**Authors:** Julius Grote, Julia Elina Stocker, Jens Sommer, Anna-Maria Hamm, Henrik Kessler, Andreas Jansen

**Affiliations:** 1 Laboratory for Multimodal Neuroimaging, Department of Psychiatry and Psychotherapy, University of Marburg, Marburg, Germany; 2 Core-Facility Brainimaging, Faculty of Medicine, University of Marburg, Marburg, Germany; 3 Department of Psychosomatic Medicine and Psychotherapy, University of Marburg, Campus Fulda, Fulda, Germany; 4 Center for Mind, Brain and Behavior (CMBB), University of Marburg and Justus Liebig University Giessen, Marburg, Germany; 5 Graduate Training Center for Neuroscience (GTC), University of Tübingen, Tübingen, Germany

**Keywords:** Tetris, fMRI, PsychoPy, Visuospatial working memory, Mental imagery, Cognitive paradigm

## Abstract

Tetris is a widely used computer game, not only for entertainment purposes but increasingly in cognitive and clinical neuroscience research. Despite its frequent application in experiments, the neural mechanisms underlying gameplay remain insufficiently understood. Prior work suggests that cognitive control during complex visuospatial tasks, like Tetris, engages the attention network as well as regions supporting visuospatial working memory, mental imagery, and motor planning. However, researchers currently lack a standardized paradigm to investigate these processes. To address this gap, we introduce *Tetrix,* a novel and flexible Tetris-based paradigm designed for use in behavioral and functional magnetic resonance imaging (fMRI) experiments. With a broad range of customizable features, *Tetrix* can be adapted to specific experimental requirements or used in its default configuration. To validate the paradigm’s feasibility for neuroimaging applications, we conducted a proof-of-concept fMRI study with six participants. Results revealed robust bilateral activity in the frontal eye fields and posterior parietal cortex – key nodes of the dorsal attention network. Additional activation was found in the cerebellum and occipital cortex. These regions are thought to support rapid spatial orienting, the encoding and maintenance of visuospatial working memory, and motor planning. The observed activation patterns therefore align with theoretical expectations and confirm Tetrix’s utility for probing relevant cognitive functions in fMRI. Moreover, the pilot dataset and corresponding effect size maps provide a valuable resource for estimating statistical power in future research. In summary, this article introduces *Tetrix* as a standardized and validated paradigm for functional neuroimaging and beyond. It is available at: https://github.com/JuliusGrote/Tetrix_Psychopy.

**Supplementary Information:**

The online version contains supplementary material available at https://doi.org/10.3758/s13428-026-03150-6.

## Introduction

Tetris is a visually engaging video game governed by a simple set of rules. On a 20-by-10 grid, players need to stack seven uniquely shaped blocks, so-called Tetrominos or Tetriminos, as efficiently as possible. These blocks descend from the top of the grid and can be rotated or moved laterally to complete rows. When a horizontal line is filled, it disappears, allowing the rows above to shift downward. To remain in the game, one must prevent the blocks from stacking to the top of the grid. As the game progresses to higher levels, the Tetrominos drop with increasing speed, making it more difficult for a player to succeed (Tetris, 2024).

In contrast to traditional cognitive paradigms, which often isolate single processes, Tetris recruits a wide range of cognitive functions. Most prominently, it relies on spatial attention and working memory as players need to rapidly (re-)orient and keep track of game elements. Additionally, mental imagery and planning play a crucial role. Players must relate currently relevant blocks to their potential next positions on the game grid. Furthermore, the motor system must be actively involved in integrating the necessary movements to position and rotate the blocks. This is associated with the involvement of various neural networks (for more information, see Results and discussion).

This makes Tetris a rich and powerful task, demanding the interaction of all these different cognitive functions, which are lacking in more simplified tasks such as the n-back, Stroop task, Posner cueing task, or Shepard–Metzler rotation task. Because it combines this cognitive complexity with rapid skill acquisition, Tetris has been widely applied in neuroscientific research. Some studies, for instance, have used it to investigate neural efficiency (Haier et al., 1992; Rietschel et al., 2012). Repeated video game training with Tetris has been shown to reduce task-related neural responses in relevant brain regions (Haier et al., 1992). Additionally, experiments altering the game’s difficulty reveal that harder rounds elicit increased activity in networks associated with motor coordination and visuospatial processing, demonstrating that higher difficulty within the same individual and experimental session leads to a stronger neural response (Rietschel et al., 2012).

In recent years, the computer game has gained recognition in clinical neuroscience, particularly for its therapeutic applications for patients with traumatic experiences and (potential) post-traumatic stress disorder (PTSD) (Iyadurai et al., 2018; Kanstrup et al., 2021; Kessler et al., 2018). A defining feature of PTSD is the occurrence of flashbacks – vivid, involuntary recollections of a traumatic event (World Health Organization [WHO], 2022). Remarkably, studies have shown that playing Tetris, either immediately after a traumatic event (e.g., a car accident) or as part of treatment for patients with long-standing complex PTSD, can reduce the frequency of intrusive memories in the following weeks (Iyadurai et al., 2018; Kanstrup et al., 2021; Kehyayan et al., 2024; Kessler et al., 2018). Regarding the underlying neural mechanisms, researchers propose that the game disrupts the formation or reconsolidation of intrusive memories by engaging the limited resources of the visuospatial working memory and mental imagery. Holding an intrusive image in mind while concurrently being engaged in a visuospatial task causes so-called dual-task interference and is thought to weaken the emotionality of the mental image (Baddeley & Andrade, 2000). This interference may prevent the (re)consolidation of traumatic memories into long-term storage, thereby reducing their intensity and recurrence frequency (Holmes et al., 2009, 2010; James et al., 2015).

However, relatively few imaging studies have attempted to characterize neural correlates of Tetris gameplay. For instance, a study by Agren et al. (2023) used functional magnetic resonance imaging (fMRI) to investigate the neural correlates of the game while controlling for motor activity. The researchers observed the strongest fMRI responses in regions associated with visuospatial processing, such as the middle temporal gyrus and the occipital lobe – findings that align with expectations. Another experiment demonstrated that Tetris performance correlated most strongly with the results of a visuospatial working memory task, though the correlation was relatively weak (*r* = 0.33; Lau-Zhu et al., 2017). Importantly, the studies discussed so far have implemented Tetris in various ways, with different sets of controls and game manipulations. As a result, the paradigms used across studies are far from standardized, making it difficult to compare findings or draw conclusions about the underlying cognitive processes. Yet this could be achieved through a template Tetris paradigm that allows for various game adaptations.

This way, researchers cannot only change the level or speed of the game, but also the manner in which it is implemented as an experimental paradigm. In fMRI studies, for instance, this requires the inclusion of several control conditions. Besides accounting for finger movements used for Tetris’ controls (Agren et al., 2023), one should also employ a control for low hierarchical visual processing and for non-specific neural activation as part of a baseline signal. In this sense, previous studies could not reveal how the mind integrates and manages the multitude of processes required for the gameplay (Agren et al., 2023; Haier et al., 1992; Lau-Zhu et al., 2017).

In summary, Tetris represents a suitable paradigm for investigating various cognitive functions in neuroscience and, in combination with imaging techniques, their underlying neural networks. However, a comprehensive exploration of these foundations requires flexible experimental designs, such as the ability to easily manipulate the game's difficulty level or to implement specific control conditions isolating individual components (e.g., by removing effects of simple motor activity or visual processing). This flexibility becomes particularly crucial if Tetris is to be used in clinically oriented studies, for example, to examine the neural mechanisms in the treatment of PTSD. Additionally, it is beneficial to standardize Tetris implementations for comparing results across different studies.

This article is divided into two parts. In the first section, we outline the foundations of our new paradigm, *Tetrix*, and provide a comprehensive overview of the settings and features that it offers. The source code of *Tetrix* is freely available. In a second part, we demonstrate the task’s feasibility for neuroimaging by conducting an exploratory fMRI study with a small group of participants. Our results highlight *Tetrix's* capability to investigate the various cognitive functions underlying Tetris gameplay. Furthermore, we report voxel-wise effect sizes from the group-level results, laying the groundwork for accurate sample size planning in future *Tetrix*-based studies.

## Tetrix: Implementation of a Tetris-based paradigm

In preparation for a larger clinical longitudinal study, we developed a neuroscience-compatible adaptation of Tetris focusing on a versatile but easy implementation. This was achieved in three distinct steps. First, a Tetris imitation was obtained from an openly available GitHub repository (educ8s, 2024) and, second, extensively modified using Python’s Pygame library (Version 2.5.2). Finally, the game – along with instructions and control conditions – was integrated into PsychoPy (Peirce et al., 2019; Version 2024.1.1). During development, game and paradigm settings were outsourced to separate configuration files, enabling easy manipulation of task variables. The source code, along with detailed instructions, can be found in a publicly accessible GitHub repository (https://github.com/JuliusGrote/Tetrix_Psychopy). In the following, we briefly describe the technical structure of the program (Technical structure and file organization). We then discuss various features and settings (Features and settings).

### Technical structure and file organization

**Fig. 1:**
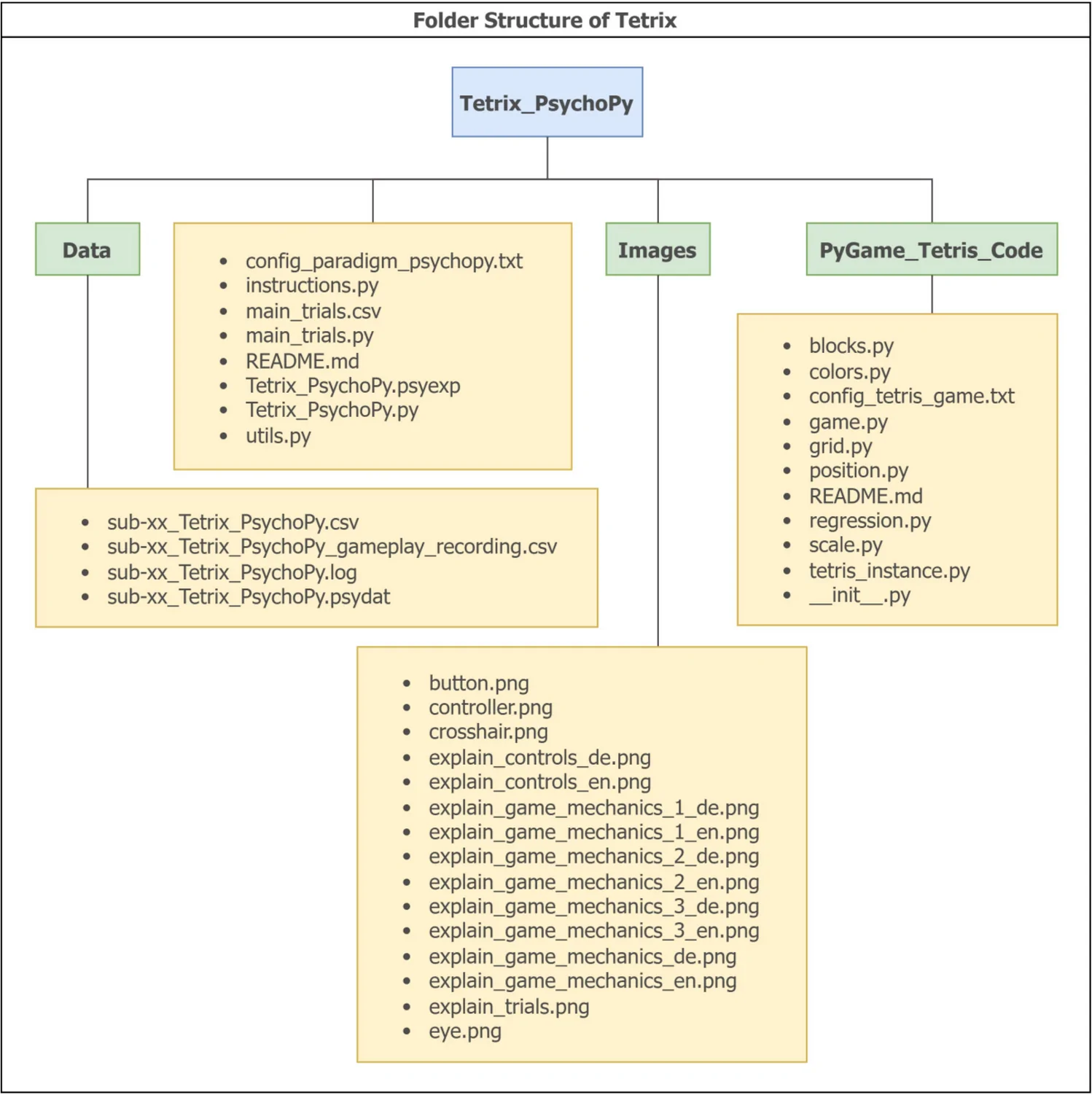
Tetrix folder structure. Green rectangles depict folders and yellow boxes list files

To understand how the program operates on a technical level, it helps to first examine its file organization (Fig. 1). The main folder, *Tetrix_PsychoPy,* contains all essential files for the PsychoPy paradigm, including the experiment file itself (*Tetrix_PsychoPy.psyexp*), scripts for generating condition orders (*main_trials.py*) and for setting instructions *(instructions.py*), as well as a collection of functions directly interacting with the operating system (*utils.py*). Within this folder, the *Pygame_Tetris_Code* subdirectory hosts Python modules responsible for the game’s core mechanics. The *Images* folder stores graphics used for explanatory routines and for cueing conditions, while the *Data* directory contains various types of log files generated by *Tetrix_PsychoPy*. Both *Tetrix_PsychoPy* and *Pygame_Tetris_Code* include configuration files. Here, *config_paradigm_psychopy.txt* holds all the design choices for the overarching Tetrix framework, whereas *config_tetris_game.txt* handles game-specific settings. Finally, the two *README.md* manuals explain the paradigm to new users.

The task itself runs as a Python script, either generated by the.psyexp file after executing a start command or directly accessed and launched from *Tetrix_PsychoPy.py*. The specific functions and modules that are subsequently called depend on the configuration of game and paradigm variables. Regardless, separate Tetris instances are created utilizing the multiprocessing package (Version 2.6.2.1; Python Software Foundation) for parallel computing:

- *pretrial_Tetris* – Assesses individual game performance (can be used as a standalone version).
- *play_Tetris* – The Tetris process used in the main trials, intended for fMRI experiments (if the main part is enabled).
- *watch_Tetris* – A visually simplified version of the game without player input, designed again for neuroimaging studies.

These processes are monitored, and proper functioning of the designated input device – such as a response box or keyboard – is validated. Depending on the current task phase or condition, some processes may run in the background, while others are resumed and brought to the foreground. In factorial task designs, the exact sequence of gameplay conditions is determined at the beginning of main trials by the *comp_wm_load_speed()* function. A more detailed description of *Tetrix’* design options will be illustrated in the next section.

To enable communication with the MR scanner, a keyboard listener records each trigger signal (converted into a specific character) throughout the experiment. Along with these timestamps, all collected variables are saved to a CSV file in the *Data* directory when the task ends.

### Features and settings

**Fig. 2:**
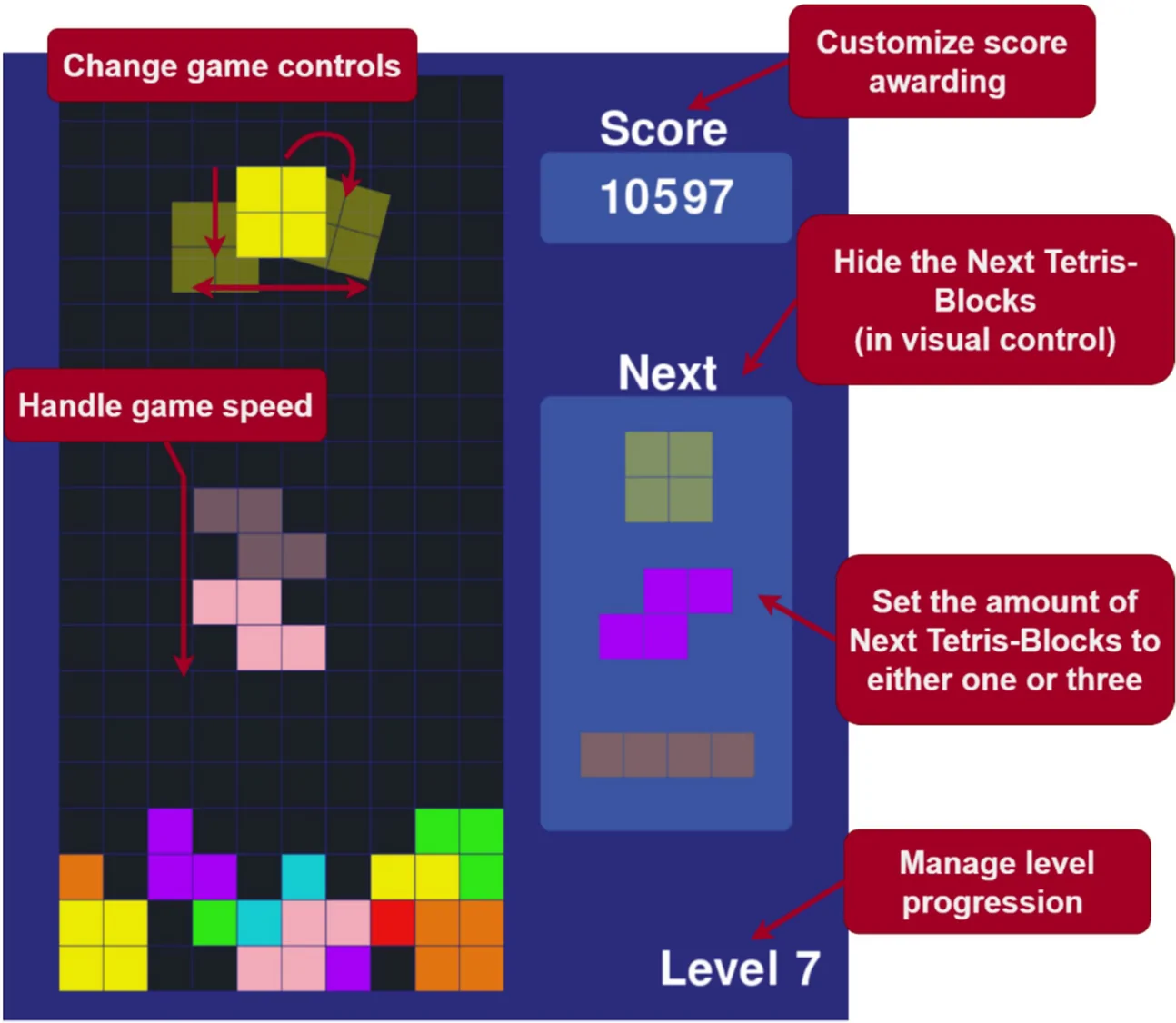
Overview of gameplay related settings for Tetrix. The interface shown here is similar to the Tetris experience during task execution. Multiple Tetrominoes are displayed simultaneously for illustrative purposes only

**Fig. 3:**
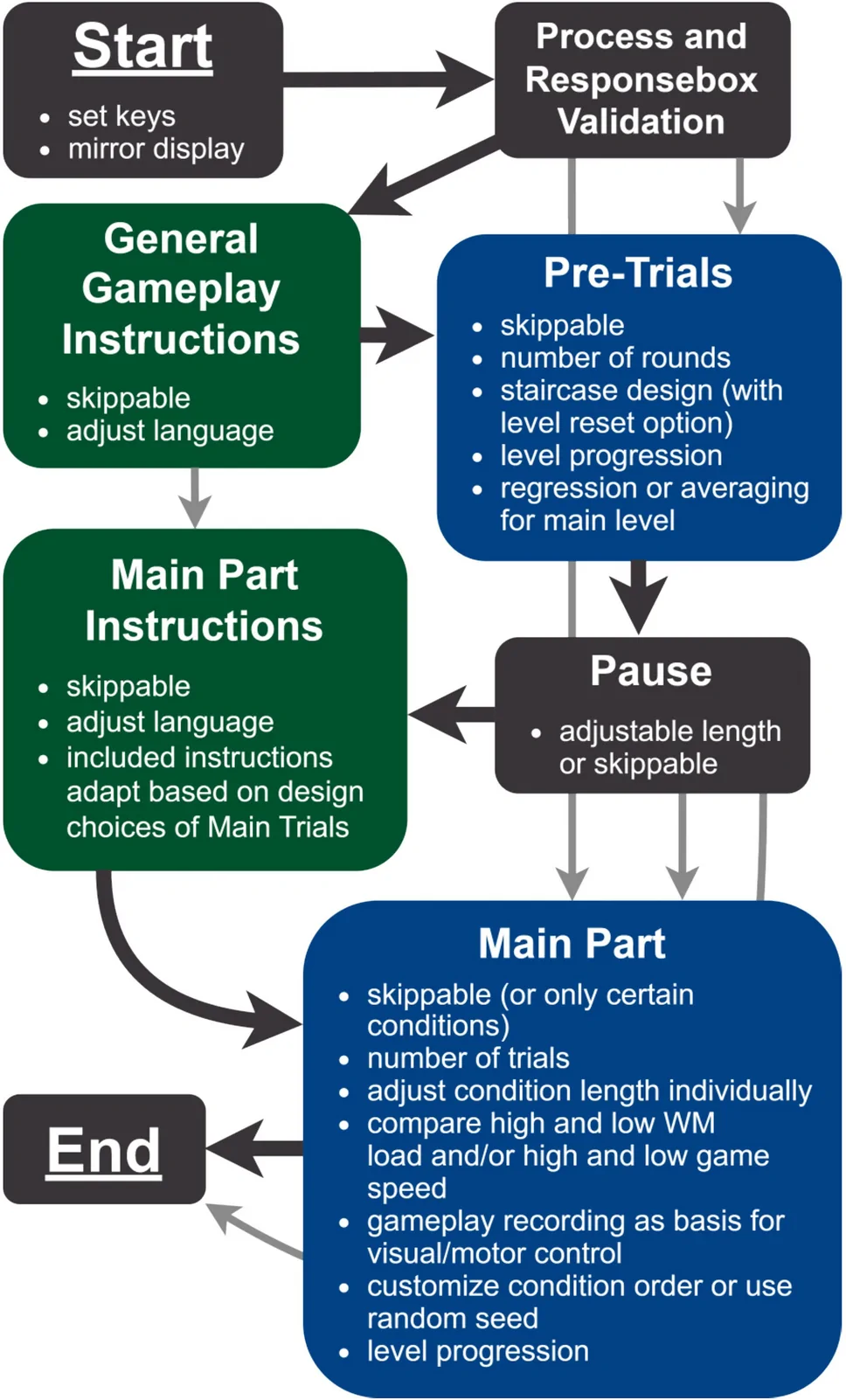
Flow chart of possible Tetrix procedures. Wide arrows represent a task procedure that utilizes the full structure of Tetrix. Narrow and lighter arrows depict alternative routes that can be taken when skipping single or multiple paradigm segments. In grey are parts mostly related to hardware interactions and core program functions. Green rectangles showcase instruction routines whereas blue ones indicate Tetris gameplay or control condition sections. Each box lists the different elements one can modify. WM working memory

*Tetrix* offers a variety of features and customization options to adapt the task to specific needs. This applies both to Tetris itself (Fig. 2) as well as to the overall paradigm structure (Fig. 3). As for the game, nearly all parameters can be adjusted. This includes defining a starting level, setting the number of lines that need to be cleared in order to reach the next level, or even disabling level progression entirely to maintain a constant difficulty level throughout the experiment. Additionally, the amount of points rewarded for clearing one, two, three, or four lines can be customized, and the speed increase per level-up can be precisely controlled.

Regarding the graphical interface, users can adjust the number of preview “*Next”* blocks, ranging from one to three, or choose to hide them entirely for the visual control condition, “*Watching Tetris”*. Moreover, *Tetrix* allows for the pseudorandomization of Tetris-block selection. This is done by generating a random seed for the function, ensuring that the Tetromino sequence remains consistent each time the task is run. After a subject loses, the score can either be reset to zero or, if enabled, kept and transferred to the next round. Similarly, the start of the next game can be automatic or require player input, meaning a key must be pressed to restart the round.

Regarding paradigm controls, users can freely customize the key bindings for in-game movements. Further, the type of downward motion can be modified. The blocks descend either by one row with a key press, are dropped completely to the bottom of the playfield, or players must hold the button to temporarily accelerate the downward movement. Integrating all these settings, the structure of *Tetrix* can be customized as well. In the Pre-Trial, users can adjust the number of Tetris rounds, the level design, and even the approach used to calculate the Tetris level for the main part (such as averaging or logistic regression).

By default, explanation routines are included but optional, allowing *Tetrix* users to disable them for purposes like testing or repeated sessions. Similarly, the break scheduled after the *Pre-Trials* has an alterable duration and, if set to zero, will be skipped entirely. Regarding the *Main Part* of the experiment, users can modify the length and number of conditions individually, and different types of “Playing Tetris” trials varying in game level or number of preview blocks can be added. Likewise, operators can configure a varying interstimulus interval with defined upper and lower boundaries.

Regarding the control conditions: In the visual control, Tetrominoes can either perform random movements with a speed based on the individual level but unrelated to player behavior, or participants watch a replay from the preceding gameplay block. Similarly, the keypress frequency, i.e., rapid symbol flashes on screen as an indication of when to press, depends by default on the *Main Part* level. Alternatively, it can mimic the exact keystroke timing of the previous playing condition or average these times to generate a uniform press frequency.

Users further have the ability to manipulate the order of conditions, which can be pseudorandomized by setting a specific seed. Each of the two experiment sections (*Pre-Trial* and *Main Part*) can be conducted independently, providing flexibility for various *Tetrix* use cases:

One group might wish to utilize the full paradigm extent for a complex fMRI analysis, while another may employ *Tetrix* as a behavioral task to assess individual Tetris skill by exclusively using the Pre-Trials. As a third option, one could use *Tetrix* as a predefined and highly demanding visuospatial exercise, perhaps in clinical trials to compare different interference tasks (similar to Holmes et al., 2010). Alternatively, some researchers may choose to focus on the MRI-related sections of the paradigm, with a fixed Tetris level for a group comparison (e.g., between age or sex).

By design, Tetrix is an ongoing project. For our pilot study, we used the original version, v1.0.0. Since then, the paradigm has been revised multiple times to implement the new functions presented in this section. Version 1.0.1 allowed each condition of the Main Part (including gameplay) to be of varying length or completely skippable. In Tetrix v1.1.0, we added detailed gameplay recording and logging of key presses during the motor control phase. Furthermore, this iteration introduced the replay settings for the visual and motor conditions described above. We will continue to provide bug fixes and new features in the future.

Because of this continuous refinement, Tetrix is versatile enough to accommodate all the aforementioned possibilities – and many more. Whether it is a quick round of Tetris as part of a larger experiment or an hour-long MRI session, *Tetrix* aims to be the new standard for Tetris applications in cognitive research and clinical settings.

## A neuroimaging pilot study

As highlighted in the introduction, we aimed to validate the feasibility of our new paradigm through an initial exploratory fMRI study. The cognitive functions we identified as relevant during Tetris gameplay were partly based on research explicitly focused on Tetris (Agren et al., 2023; Holmes et al., 2009, 2010; Lau-Zhu et al., 2017) and partly derived from experiments employing visuospatial tasks with similarities -- to the videogame (Asplund et al., 2010; Bennet & Reiner, 2022; Rahm et al., 2014; Vossel et al., 2014). Building on these studies, we expected to find activity in brain networks linked to attention processes, working memory, mental imagery and (motor) planning. When comparing Tetris gameplay to pure motor activity (via button presses), Tetris watching and baseline activity in a conjunction contrast, we predicted neural activity in various brain regions:

The lateral and medial prefrontal cortex (associated with the coordination of planning, visuospatial working memory (VSWM), and mental imagery (MI) (Christophel et al., 2017; Mattar & Lengyel, 2022; Pearson, 2019)), the cingulate cortex (particularly related to attentional processes (Petersen & Posner, 2012)), the premotor cortex and the cerebellum (both crucial for movement coordination and related state predictions (Wolpert et al., 1998; Wong et al., 2015)), as well as the posterior parietal cortex (PPC) thought to be involved in visuomotor integration and encoding of VSWM items (Christophel et al., 2017; Iacoboni, 2006).

### Methods

*Participants*: We measured seven subjects (three females, four males; mean age: 23 years, range: 21–28 years). One participant was excluded from the neuroimaging analysis due to a field-of-view shift during fMRI measurements after strong head motion, resulting in a final sample size of *N* = 6 for the investigation of functional brain images.

**Fig. 4:**
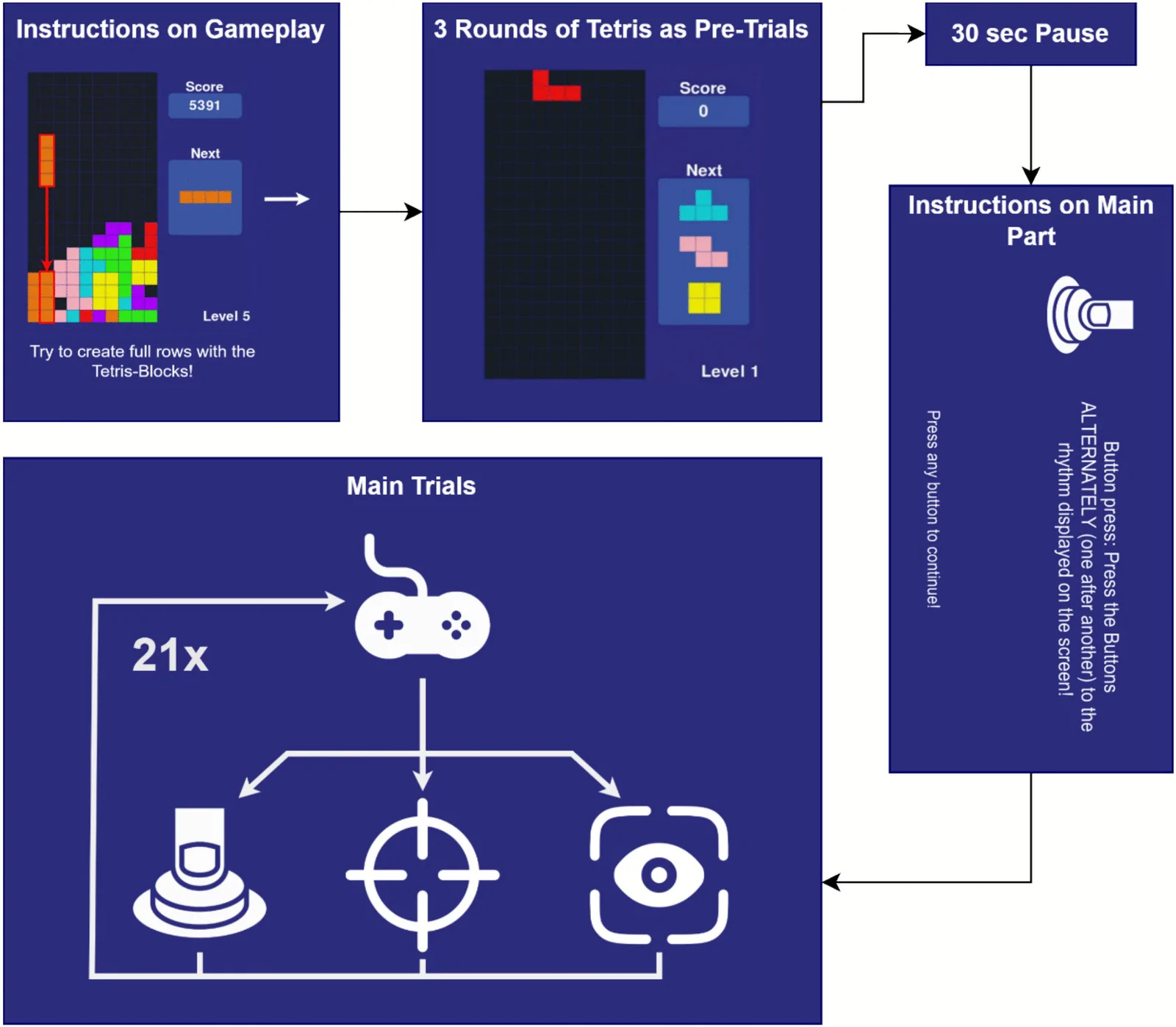
First, participants were introduced to the game mechanics and controls. They then completed three pre-trial practice rounds to calibrate the difficulty of the subsequent main trials. Following this, the conditions and structure of the main trials were explained. during the main part, each of the 21 trials began with 30 s of tetris gameplay (controller symbol). After a variable inter-stimulus interval of 6–8 s, one of three control conditions was presented according to a fixed randomized sequence: the visual control (watch tetris – *eye symbol*), the motor control (button presses – *finger/button symbol*), or the baseline condition (fixation cross – *crosshair symbol*). This sequence repeated for all trials. Functional scans were acquired exclusively during this main phase

*Experimental procedure*: The procedure went as follows. Before entering the MR scanner, participants were asked about their age, sex, previous computer game experience, and their mental fitness level on the day of the experiment. They also received explanations regarding the computer game’s rules and controls, as well as an overview of the experiment plan. To further consolidate gameplay instructions, participants had the opportunity to play one test round of Tetris. The actual experiment consisted of two parts (Fig. 4).

In the first part, the *Pre-Trial*, participants played three rounds of Tetris while being in the scanner, but no measurements were taken. The average level they reached determined the game level for the subsequent *Main Part/Main Trials*. In this section, participants encountered four different conditions. In the first condition, *Playing Tetris*, subjects were instructed to play the game normally, but they were also asked to visualize where the current and preview blocks (displayed on the right) would fit best. Importantly, level progression was disabled, and the game was paused between trials.

The second condition, the visual control condition (*Watching Tetris*), resembled actual Tetris gameplay but disabled player input. Game pieces performed random movements on the playfield and disappeared when they reached the bottom row, ensuring that the Tetris blocks did not stack on one another. Additionally, the preview “*Next*-Tetrominoes” was hidden to discourage subjects from subconsciously participating in the game (such as imagining where blocks would fit best).

In the third condition, the motor control task (*Button Presses*), participants were instructed to press the keys on the response box alternately. A symbol appeared on the monitor to indicate when to press, with a frequency based on the Tetris level. Lastly, the baseline condition consisted of a simple fixation cross in the middle of the screen.

The *Main Part* (with the actual fMRI measurements) followed a classical block design with each block lasting 30 s. A trial comprised *Playing Tetris* followed by a pseudorandomized control condition. There were 21 trials in total and the intertrial-interval varied randomly between 6 and 8 s. After completing the experiment, participants filled out another questionnaire rating the subjective difficulty and immersivity of the gameplay experience during the experiment.

*MRI data acquisition*: Participants were scanned in a 3-Tesla MR scanner (General Electric Signa Premier) with a 48-channel head matrix receive coil at the Department of Psychiatry, University of Marburg. Foam cushions and earplugs were applied to limit head motion and reduce scanner noise, respectively. A T2*-weighted single-shot gradient-echo echo-planar imaging sequence (EPI) was executed to obtain the functional images (40 slices, 3 -mm thickness with a 0.3-mm slice gap, repetition time (TR) = 1200 ms, echo time (TE) = 30 ms, multiband factor = 2, matrix size = 64 × 64 voxels, voxel size = 3 × 3 mm^2^, field of view (FoV) = 192 × 192 mm^2^, flip angle (FA) = 60°) for an acquisition time of 26 min. Whole-brain slices were acquired parallel to the intercommissural (AC-PC) plane, in descending order. Additionally, an anatomical scan using a T1-weighted rapid gradient-echo sequence in the sagittal plane (176 slices, 1-mm thickness, TR = 2368.84 ms, TE = 1.97 ms, matrix size = 256 × 256 voxels, voxel size = 1 × 1 × 1 mm^3^, FoV = 256 × 256 mm^2^, FA = 9°) was taken. This required an additional 4 min.

*Data analysis* was performed with the SPM12 software package (Wellcome Centre for Human Neuroimaging). Before preprocessing, the T1-weighted anatomical scans were skull-stripped with DeepBet (Fisch et al., 2024) and the first five functional scans were discarded. Afterward, we used MRIQC (Esteban et al., 2017), which delivers visual reports of the MRI scans together with image quality metrics (IQMs), making it easier to verify data quality. Two independent raters assessed these visual reports. Participants' data were excluded if at least one rater gave a score below 1.5.

Standard signal correction steps were performed in this order: motion estimation, outlier removal, slice time correction, co-registration, segmentation, normalization, and then smoothing. Motion estimation was achieved using a six-parameter-based affine rigid-body transformation. In order to account for head motion, we used SPIKECOR (Campbell et al., 2013), a principal component analysis-based algorithm to replace outlier volumes by interpolated values. Descending multiband slice-time acquisition of each volume was corrected by applying SPM’s Fourier phase shift algorithm, using the middle slices (*t* = 540 ms) as a reference.

Afterward, the co-registered anatomical image was segmented using the unified segmentation approach and normalized to SPM’s MNI 2 × 2 × 2 mm^3^ template. SPM used a mutual information, 12-parametric, affine regularization for estimation and transformed the images via a 4th-degree B-spline interpolation to voxel sizes of 2 × 2 × 2 mm^3^. Subsequently, all motion-corrected functional images were normalized using the transformation parameters from the previous normalization step. In the last step, these scans were smoothed with a Gaussian kernel of 8 mm to increase data sensitivity.

*Statistical modeling and inference*: We built a general linear model (GLM) with gameplay and the three control conditions as regressors convolved with the canonical hemodynamic response function. The six realignment parameters estimated from motion correction were added to the GLM as nuisance regressors, as well as the default high-pass filter that removed signals with periods longer than 128 s. The GLM estimation yielded beta estimates for each voxel in each condition. These were then contrasted using fixed-effect T-maps (family-wise error (FWE) corrected at *p* < 0.05, no voxel-count threshold) of the conjunction contrast: (Tetris > Visual Control) AND (Tetris > Motor Control) AND (Tetris > Baseline). This procedure aimed to retrieve activations associated with Tetris gameplay that were not due to motor movements, perception of basic visual cues, or unrelated default activation.

On the second level, the conjunction was achieved by a one-way ANOVA with three groups, each containing one out of three contrasts from each participant. Then, three separate contrasts with weights on each respective group were created (see Friston, 1999 and Penny, 2004 for details).

BOLD activations from the experiment were assessed both at the individual subject and the group level. As this first application of Tetrix was designed as a pilot study, we explored the data with both uncorrected and family-wise error (FWE) corrected thresholds.

The location of brain activity was assessed using both the Automatic Anatomic Labeling (AAL3) atlas Rolls et al. (2020) and the Jülich Brain Atlas provided within the SPM Anatomy Toolbox (Eickhoff et al., 2005). Compared to AAL3, the Jülich Brain Atlas offers a higher spatial resolution by delineating distinct anatomic regions based on their specific cytoarchitecture (Amunts et al., 2007). Cluster assignment was – in both cases – based on the percentage of overlap between the respective cluster and cortex parcellation.

Effect sizes were estimated by computing Hedges’ *g* (Hedges, 1981) with an approach from Gerchen et al. (2021). It involves converting voxel-wise *t*-statistics to standardized effect sizes by first computing Cohen’s *d* (Cohen, 1988) as $d=2t/\sqrt{df}$ (where df= degrees of freedom), applying a small-sample bias correction factor J=1-3/4df-1, and then multiplying to obtain Hedges’ g=d×J. Since we used a conjunction contrast for the group‑level analysis, we selected the contrast whose degrees of freedom yielded the most conservative correction factor J among the three contrasts compared.

### Results and discussion

In the following, we present the group-level activation associated with Tetris gameplay (Group level activation), provide effect size maps (Effect size estimation), and discuss data quality issues specific to the present study design (Data quality).

#### Group level activation

**Fig. 5:**
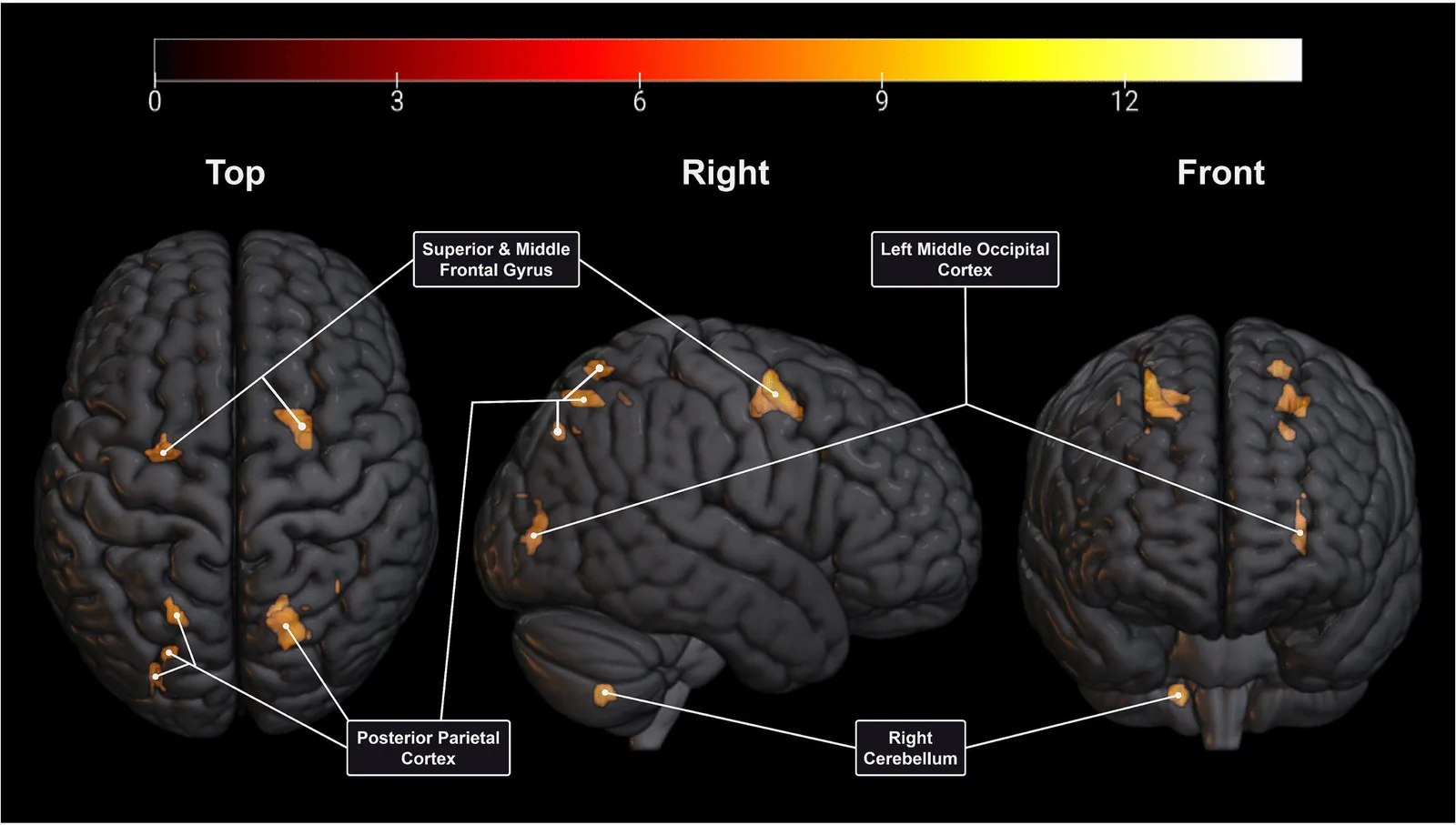
Group level activation: shown from (*left to right*) axial, sagittal, and coronal perspective. Significant clusters can be found bilaterally in the superior and medial frontal gyrus. Additional activation is located in the PPC (superior parietal lobule and interparietal sulcus), left occipital cortex and right cerebellum. The displayed T-map was FWE-corrected at *p* < 0.05

Tetris gameplay activated a distributed cortical network, with major activation clusters observed in the bilateral middle and superior frontal gyri, the bilateral posterior parietal cortex (PPC) (including the superior parietal lobule and interparietal sulcus), the left middle occipital cortex, and inferior regions of the right cerebellum (Fig. 5). A complete list of all significant clusters is provided in the supplements (Table S1). In order to replicate these group-level clusters in an independent sample, we measured an additional ten participants, showing similar results. Their group activation table can be retrieved from the supplements as well (Table S2).

The *middle and superior frontal gyri* are well known for their involvement in the preparation of complex movements – particularly those guided by external stimuli, such as visual cues that rely on learned sensorimotor associations (Ohbayashi, 2021). This process is further amplified by Tetris gameplay, which requires the mental rotation and precise positioning of falling blocks – demands that go beyond those present in the control conditions used in our study. The main activation cluster in this region was located in Area 6d3, which has also been associated with the frontal eye field (FEF) (Benjamin Marian Sigl, 2018). Although there is ongoing debate regarding the precise peak coordinates of the FEF – with different studies reporting activation in various subregions of the (lateral) dorsal frontal cortex – the location of our clusters aligns well with previous accounts in the literature (Bedini et al., 2023; Vernet et al., 2014).

The *PPC* is well known for its strong functional connectivity with the FEF (Bencivenga et al., 2023) – a relationship further supported by the co-activation of both regions observed in the present study. Together, these areas form the core of the so-called dorsal attention network (DAN), which plays a crucial role in top-down gaze control and the spatial deployment of attention (Corbetta & Shulman, 2002). Since attention and working memory are thought to rely on shared neural mechanisms (Panichello & Buschman, 2021), the observed fronto-parietal co-activation likely also contributes to the maintenance of visuospatial working memory (VSWM) items and mental imagery (MI) – which, in the context of Tetris, can be considered functionally equivalent (Curtis, 2006; Rahm et al., 2014). In this setting, the co-activation of FEF and PPC may serve a dual purpose. First, it supports the rapid (re)direction of gaze and spatial attention – allowing players to continuously select and track the most relevant elements of the game state. Second, it orchestrates the encoding, maintenance, and retrieval of attention-guided VSWM contents, such as the position and rotation state of Tetrominoes. These processes are believed to modulate activity in sensory regions, biasing processing toward task-relevant stimuli. For example, they can enhance neural activity in areas of the visual system that represent currently relevant perceptual information (Bressler et al., 2008). This may help explain why, even when visual input is controlled for, the conjunction contrast still reveals significant activation in the *middle occipital cortex*. However, it is important to note that without eye-tracking data, we cannot rule out the possibility that activity in the FEF is at least partially driven by differences in saccade frequency or distribution (Hanning et al., 2023).

Complementing the cortical network, the *cerebellum* is also known to maintain strong functional connectivity with the PPC. In concert, these regions are thought to support visuomotor integration (Iacoboni, 2006), with the cerebellum playing an additional role in generating sensory predictions of future (game) states during motor execution (Honda et al., 2018; Wolpert et al., 1998). Another potential contributor to the cerebellar activation observed in the present study could be the increased coordination demands inherent in the gameplay condition – comparable to the activity noted in the middle and superior frontal gyri.

In summary, our pilot data demonstrate that Tetris gameplay engages a distributed neural network. It activates brain regions typically associated with the diverse cognitive functions involved. Contrary to our initial expectations, we did not observe significant group-level activation in more anterior regions, such as the inferior frontal gyrus or the cingulate cortex. This absence may be attributed to the limited statistical power of the pilot dataset.

#### Effect size estimation

**Fig. 6:**
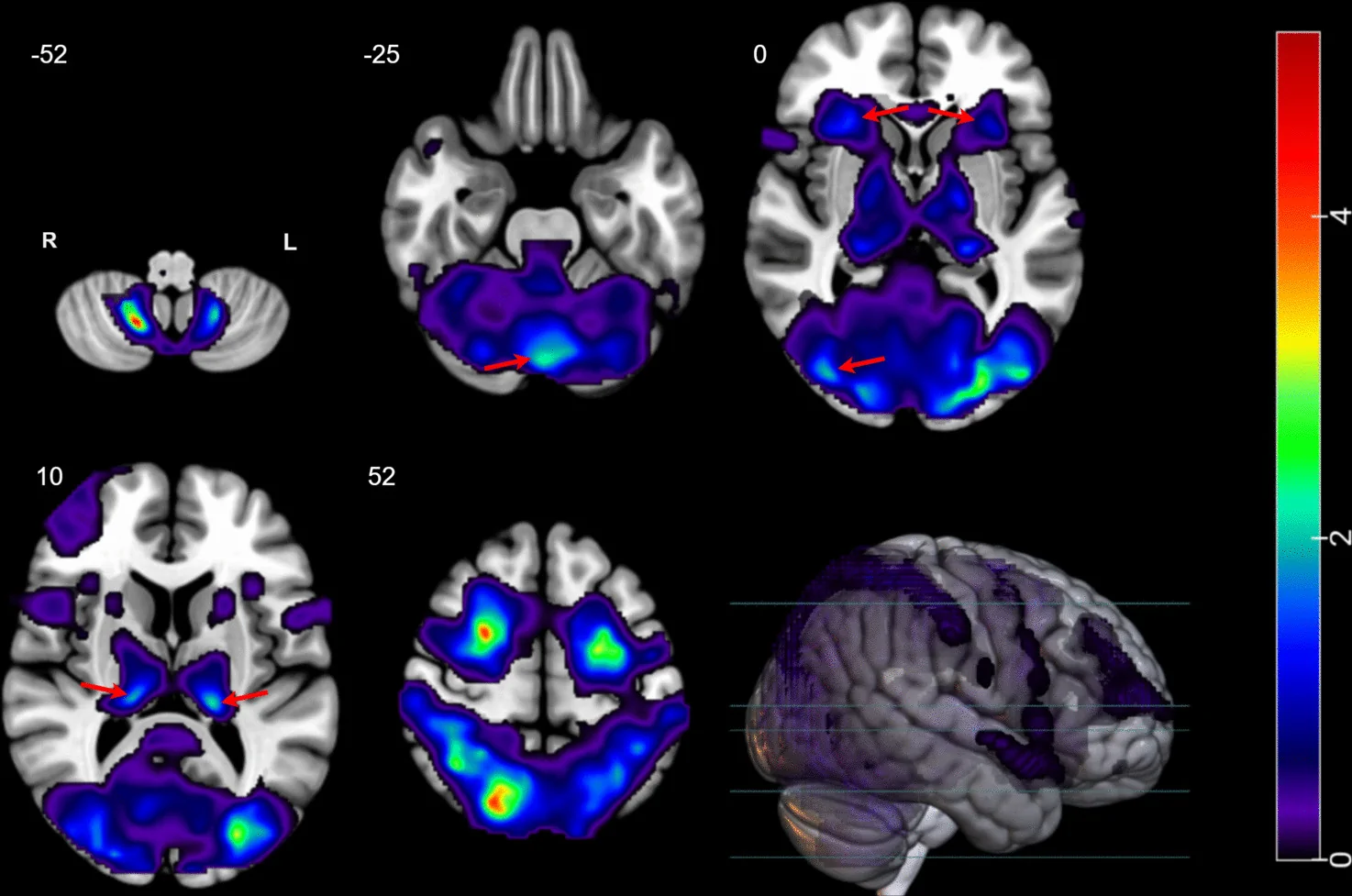
Hedges’ *g* map of the group level results. Non-colored voxels have an effect size of zero. Slices are displayed in radiological convention and shown as *dark green lines* in the rendering. *Numbers* indicate *z*-coordinates in axial direction. Besides the areas already identified in the group level comparison, comparably large effects can also be observed in the thalamus (*z* = 10), middle occipital lobe (*z* = 0), and upper cerebellum (*z* = – 25) as indicated by *red arrows*

Since the algorithm we used to compute Hedges’ *g* takes the second-level *t* values as input, the resulting effect size map (Fig. 6) closely resembles our ANOVA results. By definition, it also highlights regions that would likely reach significance given a larger sample size (red arrows Fig. 6). These include clusters in the thalamus, visual cortex, and upper cerebellum. Other prominent peak effects were observed in the anterior insula and middle frontal gyrus.

Due to the high group-level T-values and the pilot nature of the experiment (*N* = 6), some peak effect sizes were remarkably large (*g* > 5). However, pilot studies are also more susceptible to noisier and inflated effect size estimates (Sadil & Lindquist, 2024). Consequently, we observed that even non-significant areas, like parts of the occipital and temporal cortex, exhibited diffusely distributed yet strong effects. Nevertheless, these data provide a valuable basis for power calculations in upcoming *Tetrix*-based fMRI studies. This can be approached in various ways, for instance, by masking our map to hypothesis-driven regions of interest or by considering only peak effect sizes.

Through this, we address a common issue in fMRI research: inadequate sample size planning and, consequently, overestimation of effect sizes (Cremers et al., 2017; Guo et al., 2014). Additionally, Hedges’ *g* is a bias-corrected alternative to the commonly reported Cohen’s *d*, making it particularly suitable for neuroimaging studies which usually have comparably small sample sizes (Gerchen et al., 2021).

#### Data quality

In order to assess overall data quality, all raw functional scans were analyzed using MRIQC (Esteban et al., 2017). Two independent raters assigned an average score of 2.69 (scale 0–4; 4 = best data quality), indicating good data reliability. However, head motion spikes were present to varying degrees across all participants. This is likely due to the motor demands of the Tetris gameplay, which involved frequent hand and finger movements (i.e., compared to simple decision-making paradigms) and may have led to small head displacements. Even though we applied an outlier removal and interpolation algorithm, future applications of *Tetrix* in fMRI studies would benefit from further minimizing body motion inside the scanner.

## Conclusion

In this study, we introduced *Tetrix*, a novel Tetris-based experimental paradigm designed for use in neuroimaging research. Owing to its extensive customization options and flexible architecture, Tetrix can be deployed in its current form or easily adapted to accommodate new experimental and testing scenarios.

As a proof of concept, we successfully employed Tetrix in a pilot fMRI study, revealing distributed functional networks linked to attention, VSWM, and MI. While we did not observe significant group-level activity in the cingulate cortex or in prefrontal regions beyond the premotor cortex and FEF, all other hypothesized brain regions critical for cognitive control during Tetris gameplay were robustly activated.

We also observed the expected activation patterns for both control conditions (motor > baseline and watch > baseline; see Supplementary Figures S1-S2). Specifically, the watch condition elicited activity in visual processing regions (occipital fusiform gyrus, lateral occipital cortex, parahippocampal gyrus), whereas the motor condition engaged motor planning areas (superior frontal gyrus, right pallidum, cerebellum), demonstrating that the control tasks behaved as intended.

That said, these findings must be interpreted with caution due to the small sample size (*N* = 6). We do not and cannot provide strong, falsifiable predictions with these preliminary observations. Although a second independent sample of ten participants showed broadly similar activation clusters during Tetrix gameplay (see Supplementary Table S2), the current evidence remains insufficient to draw firm conclusions about the functional roles of the brain regions recruited during Tetris play.

While Tetrix offers extensive flexibility – allowing users to adjust condition length, number of trials, speed, etc. – we did not test the individual effect of these settings. Instead, we utilized a single, standard adaptation for all participants. This limitation prevents us from formulating construct-specific hypotheses at this stage. Future studies should systematically investigate whether manipulating gameplay parameters – such as game speed or the availability of a preview window – modulates the underlying neural activation patterns.

Traditionally, the therapeutic effects of Tetris in clinical contexts have been attributed to its occupation of VSWM resources (Holmes et al., 2009). However, the strong activation of the DAN observed in this exploratory study suggests that rapid visuospatial reorientation demands – rather than WM load alone – may place significant strain on the visuospatial processing system. This could help explain the game’s potential to interfere with the formation or recall of intrusive memories.

Future research should aim to replicate these initial findings in larger samples and further investigate the neural mechanisms underlying Tetris gameplay, capitalizing on the advanced design possibilities Tetrix provides. Now validated for use in neuroimaging settings, Tetrix offers a promising platform for advanced Tetris-based research in both healthy and clinical populations. A deeper understanding of the game’s neural underpinnings may ultimately contribute to the development of a unified model. Hopefully, this will clarify the basis of its therapeutic effect, assist the refinement of intervention protocols, and ultimately help to reduce the burden of those who suffer from PTSD and related disorders.

## Supplementary Information

Below is the link to the electronic supplementary material.

Supplementary file1 (DOCX 6485 KB)

## Data Availability

All data and other materials are available on the Open Science Framework (OSF) at https://osf.io/6hzbj/.

## Code availability

Code for data analysis and stimulus presentation can be found on the same OSF website.

## Declarations

### Open practices statement

All data, analysis procedures, and code are available on OSF at https://osf.io/6hzbj/. Our study was preregistered within this OSF project (https://osf.io/b8kr5). Data analysis matched the procedure we outlined in our preregistration. Due to the pilot nature of this study, no additional exploratory or follow-up analyses described in the preregistration were conducted.
