# Supplementary material for: Tetrix: A novel Tetris-based paradigm for neuroimaging research and clinical applications
Source: Behav Res Methods. 2026 Aug 24;58(10):279. doi: 10.3758/s13428-026-03150-6 (PMC13503407; doi:10.3758/s13428-026-03150-6)
Supplement: Supplementary file 1 [file 13428_2026_3150_MOESM1_ESM.docx]

**Supplements**

*Table S1: Overview of significant clusters. Labelling was done with both the SPM Anatomy Toolbox and the Automatic Anatomic Labelling Atlas 3. Additionally, the percent of overlap between cluster and anatomic region as defined by the atlas is given in percent. Clusters are ordered by peak T and Z values.*

*Fig. S1: Group-level Motor Control Activation. Uncorrected (p<0.01) T-map for the Motor Condition > Baseline contrast across six participants. Activation is found primarily in the Superior Frontal Gyrus, Right Pallidum, Cerebellum, Frontal Pole, Thalamus.*

*Fig. S2: Group-level Watch Control Activation. Uncorrected (p<0.01) T-map for the Visual Control Condition > Baseline contrast across six participants. Activation is found primarily in the Occipital Fusiform Gyrus, Parahippocampal Gyrus, Lateral Occipital Cortex, Supramarginal Gyrus, Superior Temporal Gyrus, Superior Parietal Lobule, Hippocampus.*

**Replication in an additional independent Sample (N=10)**

**Conjunction Contrast: Tetrix > watch & Tetrix > motor & Tetrix > Baseline**

*Table S2: Brain Activation Clusters generated by the SPM Anatomy toolbox. Only the first 16 clusters are presented, clusters with brain activation out of atlas coverage (cluster 7 & 9) and voxel size smaller than 30 are not shown.*

**Watch contrast: Watch > Baseline**

*Table S3: Brain Activation Clusters generated by the SPM Anatomy toolbox. Only the first 5 clusters are presented, clusters with voxel size smaller than 30 are not shown.*

**Motor contrast: Motor > Baseline**

Table S4: Brain Activation Clusters generated by the SPM Anatomy toolbox. Only the first 8 clusters are presented, clusters with voxel size smaller than 30 are not shown.

*Table S SEQ Table_S \* ARABIC 4: Brain Activation Clusters generated by the SPM Anatomy toolbox. Only first 8 clusters are presented, clusters with voxel size smaller than 30 are not shown.*
